# Factors associated with use and non-use of the Fecal Immunochemical Test (FIT) kit for Colorectal Cancer Screening in Response to a 2012 outreach screening program: a survey study

**DOI:** 10.1186/s12889-015-1908-x

**Published:** 2015-06-11

**Authors:** Nancy P. Gordon, Beverly B. Green

**Affiliations:** Division of Research, Kaiser Permanente Medical Care Program, 2000 Broadway, Oakland, CA 94611 USA; Group Health Research Institute, Group Health Cooperative, Seattle, WA USA

**Keywords:** CRC, Colorectal cancer screening, Fecal immunochemical test, Patient attitudes

## Abstract

**Background:**

The one-sample fecal immunochemical test (FIT) is gaining popularity for colorectal cancer (CRC) screening of average-risk people. However, uptake and annual use remain suboptimal.

**Methods:**

In 2013, we mailed questionnaires to three groups of nonHispanic White, Black, and Latino Kaiser Permanente Northern California (KPNC) members ages 52–76 who received FIT kits in 2010–2012: Continuers did the FIT all 3 years; Converts in 2012, but not 2010 or 2011; and Nonusers in none of the 3 years. The questionnaires covered social influences, perceived CRC risk, reasons for using (Continuers, Converts) or avoiding using (Nonusers) the FIT, and recommendations for improving the kit.

**Results:**

Continuers (*n* = 607, response rate 67.5 %), Converts (*n* = 317, response rate 35.6 %), and Nonusers (*n* = 215, response rate 21.1 %) did not differ in perceived risk or family history of CRC, but Nonusers were less likely than Continuers and Converts to know someone who had polyps or CRC. Continuers, Converts, and Nonusers did not differ in social network encouragement of CRC screening, but did differ in believing that it was very important that they be screened (88.3 %, 68.4 %, 47.7 %) and that their medical team thought it very important that they be screened (88.6 %, 79.9 %, 53.9 %). Approximately half of Continuers and Converts completed the FIT to please their doctor. Converts were less likely than Continuers to use the FIT to “make sure they were OK” (53.7 % vs. 72.6 %) or “protect their health” (46.1 % vs. 76.4 %). Nearly half of Converts completed the FIT out of guilt. Approximately half of FIT kit users suggested adding a disposable glove, extra paper, and wider-mouth tube to the kit. Nonusers’ reasons for not using the FIT included discomfort, disgust, or embarrassment (59.6 %); thinking it unnecessary (32.9 %); fatalism/fear (15.5 %); and thinking it too difficult to use (14.5 %), but <10 % did not want CRC screening at all.

**Conclusions:**

Nonusers and irregular users of the FIT are less intrinsically motivated to get CRC screening than long-term users and more averse to preparing their stool sample. Changes to the FIT kit to address discomfort and difficulty factors might improve uptake and continued use.

**Electronic supplementary material:**

The online version of this article (doi:10.1186/s12889-015-1908-x) contains supplementary material, which is available to authorized users.

## Background

Colorectal cancer (CRC) is the third leading cause of cancer deaths in the United States [1]. The United States Preventive Services Task Force has recommended that adults ages 50–75 be screened for CRC by one of three evidence-based methods: an annual high-sensitivity fecal occult blood test (FOBT) , flexible sigmoidoscopy every five years, or colonoscopy every ten years [2]. Yet, on average, only 65 % of Americans are meeting recommended guidelines for CRC screening [3]. Patient-reported barriers to CRC screening include failure of physicians to recommend screening, scheduling difficulties, cost, lack of insurance coverage, gaps in knowledge, fear, embarrassment, pain, aversion, lack of symptoms, and perceived low risk [4–9].

Many of these previous studies have focused on sigmoidoscopy and colonoscopy for CRC screening. Some patients who have undergone or know someone who has undergone CRC screening describe these methods as invasive, uncomfortable, and inconvenient because they must be done in a medical setting and require a colonoscopy bowel preparation prior to the procedure. FOBTs and the newer fecal immunochemical test (FIT) are recommended to be done annually rather than every ten years, but in contrast to colonoscopy, are done at home and involve no special preparation. Studies have shown that offering the newer FIT, which involves no dietary restrictions prior to the test and requires preparation of only one stool sample (versus three for the FOBT), results in higher uptake than the FOBT [10–15]. However, despite the convenience factor, uptake of the FIT is still suboptimal (50–70 %).

Reasons for not completing the stool-based CRC screening tests (primarily the FOBT) include feeling healthy, believing oneself to be at low risk, having no CRC symptoms, fearing results of a positive screening test requiring a follow-up colonoscopy, and not wanting to know if CRC is present [5, 16–21]. Barriers specific to the test itself include difficulty understanding the instructions for how to take and prepare the stool sample; concerns about handling, storing, and mailing the stool sample; concern about test accuracy; and a preference for a doctor to do such tests [5, 8, 22–24].

In 2005, in an effort to boost CRC screening rates, the Kaiser Permanente Medical Care Program in Northern California (KPNC) implemented a centralized outreach effort to promote CRC screening for members at average risk using mailed FIT kits [25]. Strategic enhancements to encourage use of the kit (e.g., test was free for all members, letter from primary care provider, multiple reminders, and multiple kit mailings) resulted in 68 % of the 500,500 member outreach cohort ages 50–75 (65 % of 50–64 year olds and 78 % of 65–75 year olds) completing the FIT in 2012.

To identify steps that could be taken to increase uptake and annual use of the FIT and improve overall CRC screening outreach efforts, we conducted a survey of three groups of health plan members who were in FIT kit outreach cohorts for three consecutive years (2010–2012). “Continuers” were people who completed the FIT all three years; “Converts” completed the FIT in 2012, but not in 2010 or 2011; and “Nonusers” did not complete the FIT in any of these years. The aims of the survey were to learn (1) about the roles of physicians and other social influences, beliefs related to CRC screening, and other factors that motivated use or non-use of the FIT kit, and how these differed across the three groups; (2) the extent to which characteristics of the FIT kit, including modifiable components, might be affecting uptake by Nonusers and leading to irregular use among some kit users; and (3) whether there are racial/ethnic, age, or sex differences in motivations and barriers for using the FIT kit or for being screened for CRC that could inform CRC screening outreach programs.

## Methods

### Study setting

In 2012, during January to July, 500,550 health plan members ages 50–75 who were due for CRC screening were sent a computer-generated letter from their primary care doctor telling them that the FIT kit was coming, why it was important to get screened for CRC, and that the test was free to all members. The FIT kit (Polymedco OC FIT-CHEK®) arrived at their home approximately one week later with a multilingual flyer about the importance of being screened for CRC and a pre-paid cardboard mailer to send the stool sample to the processing lab. Members who did not send in their completed FIT sample received an automated phone reminder approximately four weeks after the mailing and a follow-up letter two weeks after that. During August to October, those who had not yet completed the FIT were sent a second FIT kit. In addition, medical facilities were encouraged to implement CRC screening “inreach” activities, offering members whose electronic health record showed they were past due for CRC screening a FIT kit at primary care visits or flu shot clinics. Among English speakers (the only language group included in this study), approximately 71 % of nonHispanic Whites (WhiteNH), 66 % of Blacks, and 64 % of Latinos in the 2012 outreach cohort completed the FIT.

### Study sample

The study population included three groups of health plan members ages 52 to 76 who received mailed FIT kits in three consecutive years (2010, 2011, and 2012). The study groups were retrospectively created by identifying 2012 FIT completion status and then looking back at 2010 and 2011 completion status. “Continuers” completed the FIT in all three years; “Converts” completed the FIT in 2012, but not in 2010 or 2011; and “Nonusers” did not complete the FIT in any of the three years. The three study groups were linked with member race/ethnicity and language preference data derived from a combination of clinical, administrative, and research data sources; about 99 % had language preference data, and among those whose language preference was English, 98 % of Continuers, 96 % of Converts, and 91 % of Nonusers were matched to a race and ethnicity. From the identified WhiteNH, Black, and Latino English speakers who were still current health plan members, we selected stratified random samples of 900 Continuers, 891 Converts, and 1020 Nonusers. All three study groups included approximately equal numbers of WhiteNH, Black, and Latino men and women who were ages 52–64 or 65–76 at the time of the survey.

### Data collection

Questionnaire items were derived from previously published results of focus group and survey research about factors associated with use or avoidance of the FOBT [5, 26–31] and results of qualitative interviews conducted with KPNC members in 2012. Most questions employed a checklist format that also allowed respondents to write in their own free-text responses.

Continuers and Converts received the same brief (3-page) questionnaire, and Nonusers received a different questionnaire that had many items overlapping with the Continuer/Convert questionnaire (see Additional file 1). The print survey materials were mailed in August 2013, with a second survey packet sent to nonrespondents approximately five weeks later. The survey could also be completed online. Participants were offered a $10 gift card for completing the survey. This study was approved by the Kaiser Foundation Research Institute’s Institutional Review Board.

### Data analysis

Respondent data were weighted back to the race/ethnicity (WhiteNH, Black, and Latino) × age group (52–64, 65–76) × sex strata of the study population Continuer, Convert, and Nonuser groups from which they were sampled. All analyses were performed using SAS version 9.3 [32]. Weighted percentages were produced by using SAS Proc Surveymeans. SAS Proc Surveylogistic was used to test for significant differences between study groups after adjusting for the demographic factors using multivariable models that included race/ethnicity (Black, Latino vs. WhiteNH), age group (65–76 vs. 52–64), sex (female vs. male), and study group (Convert vs. Continuer or Convert, Nonuser vs. Continuer). A Wald chi-square value with *P* < .05 was considered statistically significant. All comparisons cited as statistically significant in the text met the *P* < .05 threshold.

## Results

### Survey response rate and exclusions

The response rate after two mailings was 67.5 % (607/899) for the Continuer group, 35.6 % (317/890) for the Convert group, and 11.7 % (119/1017) for the Nonuser group, although the Nonuser sample was increased to 21.1 % (215/1017) with the addition of data from an abbreviated phone interview with a sample of 100 nonrespondents. Across all three groups, there was no difference in response by age (52–64 vs. 65–76), but WhiteNHs were slightly more likely to respond than Blacks and Latinos (see Additional file 2). After data collection, 5 Continuers, 3 Converts, and 1 Nonuser were excluded when we found that they were not in of any the three racial/ethnic groups being studied; 1 Continuer, 1 Converts, and 3 Nonusers were removed from the original survey sample during data collection due to leaving the health plan (*n* = 1) or unavailability of a current mailing address (*n* = 4).

### Demographic and health characteristics of the study groups

The final Continuer, Convert, and Nonuser respondent groups were very similar with regard to age, sex, and racial/ethnic composition prior to applying the weighting factor (not shown). Table 1 shows a comparison of these groups using weighted data. Compared to the Continuer group, the Convert and Nonuser groups had significantly higher percentages of 52–64 year olds and non-WhiteNH. Continuers and Converts were similar with regard to education, family history of CRC, personal history of any type of cancer, daily low-dose aspirin use, and most of the dietary practices. However, Converts were significantly less likely than Continuers to have a history of irritable bowel syndrome (*P* < .05) and to say they ate a lot of high-fiber foods or took a fiber supplement (*P* < .001). Compared with Continuers and Converts, Nonusers were significantly less likely to have attended any college (*P* < .001) and significantly less likely to know someone with a history of colon polyps or CRC (*P* < .001).

**Table 1:** Characteristics of respondents (after weighting)

	Continuers + Converts	Continuers	Converts	Nonusers
	(*N* = 916)	(*N* = 602)	(*N* = 314)	(*N* = 214)^1^
	Wtd. % (n)	Wtd. % (n)	Wtd. % (n)	Wtd. % (n)
Group (% Continuers)	90.6 (602)	100 (602)	--	--
Sex and age				
Female 52–64	38.0 (207)	35.0 (123)	43.7 (84)	41.4 (48)
Male 52–64	31.8 (202)	29.3 (140)	36.6 (62)	37.7 (46)
Female 65–76	17.0 (249)	20.0 (163)	11.2^*^ (86)	11.9^*^ (60)
Male 65–76	13.2 (258)	15.7 (176)	8.5^*^ (82)	9.0^*^ (60)
Race/ethnicity				
nonHispanic White	82.4 (343)	83.0 (220)	76.7^*^(123)	76.2^*^ (74)
Black	8.1 (276)	7.8 (182)	10.3 ( 94)	10.3 (67)
Hispanic/Latino	9.5 (297)	9.1 (200)	13.0 ( 97)	13.5 (73)
Education				
< 12 years	2.3 ( 54)	2.3 ( 34)	2.5 ( 20)	3.9 ( 11)
High school graduate/GED	15.7 (204)	15.4 (146)	18.2 ( 58)	25.5 ( 64)
Some college	36.3 (348)	36.4 (232)	35.3 (116)	30.9 ( 79)
College graduate	45.7 (329)	45.9 (214)	44.0 (115)	39.7^*^ (56)
In a committed relationship	78.7 (647)	79.5 (439)	70.7 (208)	73.2 (143)
Other				
Personal history of cancer	11.1 ( 83)	11.3 ( 59)	8.6 (24)	7.8 (20)
Family history of CRC	12.1 ( 99)	12.3 ( 63)	10.2 (36)	7.7 (15)
Knows someone who had CRC or pre-cancerous polyps	35.9 (272)	36.3 (186)	32.5 (86)	29.4^*^ (40)
Recalls getting more than 1 FIT kit in the mail in 2012	30.8 (225)	12.4 ( 67)	54.7 (158)	57.3 ( ^a^ )

*Wtd* weighted, *GED* general education development (high school equivalency exam), *CRC* colorectal cancer, *FIT* fecal immunochemical test

^*^Significantly different (*P* < .05) from Continuer group after adjusting for age group and race/ethnicity

^a^Not asked in the abbreviated interview

^1^Data for Nonusers comes from 118 people who completed the questionnaire and 96 people who completed a phone interview that asked a subset of the questions. Demographic characteristics for the two groups did not differ significantly

### Differences in physician and social network influences on use of the FIT kit

Converts and Nonusers were significantly more likely than Continuers to indicate that their doctor had discussed CRC screening in general or specifically the FIT with them (52.6 % and 62.4 % vs. 35.1 %, respectively). This was not unexpected, because physicians were asked to discuss CRC screening with patients who were past due for screening as part of the outreach strategy. There were no significant differences between Continuers and Converts in the types of people who encouraged them to get CRC screening (Table 2). However, across all three groups, men were significantly more likely than women to identify at least one person in their social network who had encouraged them to get CRC screening. Among men, this was most frequently their spouse/partner. Sources of encouragement among women tended to be other relatives or friends. Very few people indicated getting encouragement from employers or religious leaders (ministers, priests, or rabbis). Nonusers did not significantly differ from Converts with regard to having discussed CRC screening with their doctor and other sources of encouragement, and there were no significant racial/ethnic differences in social network encouragement for CRC screening.

**Table 2:** Responses to the question “Aside from your doctor, who has encouraged you to get screened for CRC?”

	Continuers		Converts		Nonusers	
	Men	Women	Men	Women	Men	Women
	(*n* = 314)	(*n* = 286)	(*n* = 142)	(*n* = 309)	(*n* = 106)	(*n* = 108)
	Wtd. %	Wtd. %	Wtd. %	Wtd. %	Wtd. %	Wtd. %
Any family member	58.2	33.8^*^	65.7	41.6	67.6	35.9^*^
Spouse/partner						
All	50.9	23.1^*^	57.3	23.5^*^	64.8	26.9^*^
Those with a spouse/partner	59.6	31.5^*^	70.3	36.5^*^	76.5	37.9^*^
Children/grandchildren	4.3	5.0	2.7^**^	11.1^*^	2.1	8.3^*^
Other relatives	10.7	12.9	10.6	11.8	6.6	15.7^*^
Friends or co-workers	8.6	15.1	17.1	19.4	3.1	12.9^*^
Employer	0.1	0.1	1.8	2.8	0	7.9
Minister, priest, or rabbi	0.1	0.1	0.5	0.3	0	3.6
None of these relationships	39.8	57.2^*^	29.1	45.6	28.8	50.0^*^

*Wtd* weighted, *CRC* colorectal cancer

^*^Significant (*P* < .05) difference between the sexes in the same group, after adjusting for age group and race/ethnicity

^**^Significant (*P* < .05) difference between Converts and Continuers within sex group after adjusting for age group and race/ethnicity

### Differences in beliefs about personal CRC risk and importance of getting screened

#### Perceived level of risk for developing CRC

There was no significant difference in perceived risk of developing CRC across the three groups. Approximately 60 % of adults believed they were at very low or low risk, about one-fourth thought they were at medium or high risk, and the rest were not sure of their risk (see Additional file 3).

#### Belief that the respondent’s medical care team thinks it is very important for the respondent to get screened

Continuers (88.6 %) were significantly more likely than Converts (79.9 %), who were significantly more likely than Nonusers (53.9 %, based on questionnaire responders only) to think that it is very important to their medical care team that they get screened for CRC (see Additional file 4), and these significant group differences remained after adjusting for age group, race/ethnicity, and sex. Although belief about importance to the medical care team did not significantly differ by sex or age group, the Continuer-Convert difference was statistically significant for Whites (88.2 % vs. 75.4 %) and Latinos (92.3 % vs. 79.4 %), but not Blacks (88.7 % vs. 83.8 %). Racial/ethnic differences in the Nonuser group could not be studied due to the small size of the subgroups. However, after adjusting for race/ethnicity, we found no significant age group or sex differences in beliefs about the importance of screening to subjects’ medical teams.

#### Personal belief that it is very important to get screened

Continuers (86.8 %) were significantly more likely than Converts (68.4 %), and Converts were significantly more likely than Nonusers (47.4 %) to think it is very important that they get screened for CRC (see Additional file 5). The difference in percentages of Continuers and Converts holding this belief was statistically significant for WhiteNHs (85.9 % vs. 65.9 %), Blacks (95.2 % vs. 84.6 %), and Latinos (88.3 % vs. 69.6 %). For both Continuers and Converts, Blacks were significantly more likely than WhiteNHs to think that getting CRC screening was very important.

Logistic regression models run separately for Continuers, Converts, and Nonusers found a significantly positive association between believing that it is very important to get screened and believing that one’s medical care team thinks it is very important to be screened (Continuers: *χ*^2^ = 34.5, *P* < .001; Converts: *χ*^2^ = 23.8, *P* < .001; Nonusers: *χ*^2^ = 24.6, *P* < .001) after adjusting for sex, age group, and race/ethnicity. Similar models found a significant negative association between belief that it is very important to get screened and belief that one does not have a medium or high risk of developing CRC (Continuers: *χ*^2^ = 4.7, *P* < .05; Converts: *χ*^2^ = 7.2, *P* < .01; Nonusers *χ*^2^ = 10.6, *P* < .01). When belief about personal CRC risk and belief about importance to one’s clinicians were both entered into the logistic model, the latter remained strongly significant for all three groups. Perceived CRC risk remained statistically significant (*χ*^2^ = 5.5, *P* < .05) for Continuers and Nonusers (*χ*^2^ = 5.5, *P* < .02), but not for Converts (*χ*^2^ = 2.2, *P* > .10).

#### Awareness of need to complete the FIT annually for effective screening

Converts and Nonusers were significantly less likely than Continuers to know that the FIT needs to be done every year to be effective for CRC screening (72.8 % and 42.9 % vs. 90.4 %, respectively).

#### Awareness of time frame for use of the FIT kit and sending in the sample

The FIT kit has a shelf life of about ten months, but we found that across all three study groups, approximately 75 % of people thought that the FIT kit needed to be used within four weeks of receipt. Continuers were significantly more likely than Converts to think that the test needs to be completed within two weeks (56.6 % vs. 30.9 %, *χ*^2^ = 18.8, *P* < .0001). Also, unless the ambient air is very warm, the stool sample can be processed up to fourteen days after being put in the tube, meaning that it could be mailed or dropped off 7–8 days after the sample is prepared. We found that 85 % of Continuers and Converts thought that their sample needed to be in the mail within three days, and 95 % thought within five days; among Nonusers, 70 % thought it needed to be mailed within five days and nearly 25 % thought it was OK to mail it ten days later.

### Reasons indicated by Continuers and Converts for completing the FIT

Over half of men in the Continuer and Convert groups and half of women in the Convert group said they completed the FIT because their doctor really wanted them to, but women in the Continuer group were significantly less likely than men in that group to say this (*χ*^2^ = 6.19, *P* < .02, after adjusting for age and race/ethnicity) (Table 3). In both groups, Black men were significantly more likely than WhiteNH men (*χ*^2^ = 4.5, *P* < .05) to say they did the FIT because their doctor wanted them to, but no significant racial/ethnic differences were observed for women.

**Table 3:** Reasons indicated by Continuers and Converts for completing the FIT in 2012

	Continuers	Converts	Continuers		Converts	
	All	All	Men	Women	Men	Women
	(*N* = 600)	(*N* = 308)	(*N* = 315)	(*N* = 285)	(*N* = 138)	(*N* = 170)
	Wtd. %	Wtd. %	Wtd. %	Wtd. %	Wtd. %	Wtd. %
People who really wanted me to do it						
My doctor	44.2	54.0	52.4	37.4^*^	58.5	50.5
My spouse/partner						
All	19.4	23.2	33.4	7.8^*^	41.8	8.5 ^*^
Those in a committed relationship	24.6	34.1	38.9	10.8^*^	53.2	13.9^*^
My children/grandchildren	3.7	6.9	5.2	2.4	2.9	10.1^*,**^
I know someone with a history of CRC or colon polyps	27.3	20.1	17.8	35.2^*^	14.7	24.5
Peace of mind/prevention						
I wanted to make sure I was OK	72.6	53.7^**^	68.9	75.7	51.2 ^**^	55.7^**^
FIT test can help me protect my health	76.4	46.1^**^	70.5	81.3^*^	38.5 ^**^	52.1^**^
When colorectal polyps are found and removed, CRC can be prevented	41.3	22.7^**^	36.0	45.8	24.9	21.1^**^
FIT is more convenient than other CRC screening methods	66.2	41.0^**^	59.9	71.4^*^	35.2^**^	45.5^**^
Felt guilty after receiving so many kits						
All	4.6	47.2^**^	1.8	7.0	37.9^**^	54.5^**^
Those who recalled getting >1 kit^a^	12.3	65.3^**^	0.7	12.7^*^	68.9^**^	63.4^**^

*Wtd* weighted, *FIT* fecal immunochemical test, *CRC* colorectal cancer

^*^Significant (*P* < .05) difference between sexes within Continuer or Convert group after adjusting for age group and race/ethnicity

^**^Significant (*P* < .05) difference between Convert and Continuer group within sex group after adjusting for age group and race/ethnicity

^a^43 (13.6 %) men and 59 (20.7 %) women in the Continuer group reported getting multiple FIT kits whereas 69 (50.0 %) men and 114 (67.0 %) women in the Convert group reported getting multiple kits

In both groups, men were significantly more likely than women to indicate that they did the FIT because their spouse/partner really wanted them to (All: *χ*^2^ = 30.3, *P* < .001; those in a committed relationship: *χ*^2^ = 24.5, *P* < .001; models adjust for age group and race/ethnicity). Among those in the Convert group who were in a committed relationship, Blacks were significantly more likely than WhiteNHs to indicate spouse/partner as an important influence (Women: *χ*^2^ = 7.2, *P* < .01; men *χ*^2^ = 3.9, *P* < .05). In both groups, women were significantly more likely than men (*χ*^2^ = 11.0, *P* < .001) to indicate that knowing a family member or friend who had CRC or colon polyps motivated them to do the FIT.

In both groups, personal reasons were more frequently endorsed than social influences as motivating FIT kit use. However, after adjusting for age, sex, and race/ethnicity, Continuers were significantly more likely than Converts to indicate that they did the FIT to protect their health (76.4 % vs. 46.1 %, *χ*^2^ = 33.2, *P* < .0001), to make sure they were OK (72.6 % vs. 53.7 %, *χ*^2^ = 14.0, *P* < .001), and because CRC can be prevented if polyps are found and removed (41.3 % vs. 22.7 %, *χ*^2^ = 11.9, *P* < .001). Among both Continuers and Converts, Blacks were significantly more likely than WhiteNHs to say they had done the FIT to make sure they were OK (*χ*^2^ = 7.8, *P* < .01).

Continuers were significantly more likely than Converts to indicate that they used the FIT kit because it was more convenient than other CRC screening methods (66.2 % vs. 41.0 %, *χ*^2^ = 20.3, *P* < .001). However, among the Continuers, WhiteNHs were significantly more likely than Blacks and Latinos to indicate convenience of the FIT as a reason for doing it.

Converts were significantly more likely than Continuers to be motivated to do the FIT out of guilt from receiving so many FIT kits (47.2 % vs. 4.6 %, *χ*^2^ = 66.4, *P* < .001). Furthermore, 9 % of women in the Convert group indicated guilt as the *only* reason they did the test.

After indicating all their reasons, participants were asked to indicate their top two reasons. The results are shown in Table 4. “Wanting to make sure I was OK” was a top reason for both Continuer and Convert groups, as well as for all races/ethnicities and both sexes. Doing the FIT to help protect health ranked higher among Continuers than Converts. Very low percentages of both groups (7 % of Continuers and 6 % of Converts) ranked doing the test for early detection and removal of polyps as a top reason. Wanting to please their doctor was a top reason for men and women in the Convert group and men in the Continuer group. Doing the FIT to please their spouse was a top reason for a higher percentage of men in the Convert than Continuer group, and doing it to please children was endorsed as a top reason by no men and <2 % of women. Guilt about having received multiple kits was a top reason for approximately one-fourth of Converts overall and over one-third of those who recalled receiving multiple kits.

**Table 4:** Reasons most frequently cited as one of the top two motivations for doing the FIT in 2012

	Continuers	Converts	Continuers		Converts	
	All	All	Men	Women	Men	Women
	(*N* = 600)	(*N* = 308)	(*N* = 315)	(*N* = 285)	(*N* = 138)	(*N* = 170)
	Wtd. %	Wtd. %	Wtd. %	Wtd. %	Wtd. %	Wtd. %
My doctor really wanted me to do it	18.9	28.3^*^	25.1	13.7^**^	25.9	30.2^*^
My spouse/partner really wanted me to do it (those in a committed relationship)	7.1	15.4^*^	12.5	2.0	26.8^*^	3.3
I wanted to make sure I was OK	35.1	26.6	36.9	33.6	23.6	29.0
FIT test can help me protect my health	33.0	15.5^*^	30.2	35.3	7.8^*^	21.6^*,**^
FIT is more convenient than other CRC screening methods	24.5	11.8^*^	19.8	28.4	8.5	14.5^*^
Felt guilty after receiving so many kits						
All	1.1	27.0^*^	0.6	1.6	19.2^*^	33.1^*^
Those who recalled getting > 1 kit^a^	2.0	38.7^*^	0.7	2.6	34.4^*^	41.1^*^

*Wtd* weighted, *FIT* fecal immunochemical test, *CRC* colorectal cancer

^*^Significant (*P* < .05) difference between Convert and Continuer group within sex group after adjusting for age group and race/ethnicity

^**^Significant (*P* < .05) gender difference within Continuer or Convert group after adjusting for age group and race/ethnicity

^a^43 (13.6 %) men and 59 (20.7 %) women in the Continuer group and 69 (50.0 %) men and 114 (67.0 %) women in the Convert group reported getting multiple kits

### Reasons indicated by Nonusers for not completing the FIT

Nonusers were given a list of reasons for not having done the FIT and allowed to add others. Free-text reasons that overlapped with checklist categories were recoded prior to analysis. At least one reason was given by 95 % (*n* = 204) of respondents, and these reasons were grouped into three main categories (Table 5). Approximately 60 % of Nonusers endorsed feelings of discomfort or disgust when they thought about the process of getting, preparing, and/or mailing the stool sample, or embarrassment about having anyone see that they were going to do the test. For over half (54 %), these negative feelings about the physical aspects of the FIT were the main reason for not doing it. About 33 % did not think it was necessary for them to get screened for CRC because they were feeling fine or they thought their CRC risk was very low. Approximately 12 % were extremely fearful about the test discovering cancer (“If I have colon cancer, I don’t want to know”), but only 6 % were fatalistic (“If I’m meant to get colon cancer, I will get it no matter what”; “Even if colon cancer is detected early, nothing can be done about it”). Concern about ability to get the stool sample into the tube or use the catch paper were indicated by about 15 %, and 8 % said they did not want to worry while waiting for the results. Only 4 % thought that the FIT was not going to be effective for finding cancer early.

**Table 5:** Nonusers’ reasons for not using the FIT

	*N* = 204
	Wtd. %
Feelings of discomfort, disgust, or embarrassment	59.6
The idea of doing this test involving my bowel movement makes me uncomfortable	44.3
I feel disgusted by the idea of reaching into the toilet to get the stool sample	29.5
It’s too messy to do this test	20.0
I don’t like the idea of sending my bowel movement sample through the mail	15.5
I am concerned about coming into contact with germs and bacteria	13.2
I am embarrassed to put the FIT kit by the toilet, but then I keep forgetting to use it	10.5
CRC screening not necessary	32.9
I think my chances of developing CRC are very low	27.2
I feel fine, so why look for trouble	12.8
Fatalism or fear	15.5
If I have colon cancer, I don’t want to know	12.4
I think if I am meant to get colon cancer, I will get it no matter what I do	5.3
I think that even if colon cancer is detected early, nothing can be done about it	2.9
Other	
I think it will be too hard for me to get the bowel movement sample and put it in the tube; I tried to scoop up a bit of the bowel movement and put it into the tube, but it was too hard to do	14.5
I don’t want to have to worry until I get the results of the test	8.1
I don’t want to have to pay for this test	8.0
Procrastination (e.g., “I just kept putting it off”)	7.2
I used the FIT kit to get the sample, but then forgot to put the envelope in the mail right away	6.5
I don’t think that the FIT test is effective for finding cancer early	4.2
Worried that I will need a follow-up colonoscopy and I don’t want to get one	3.1
Have problems with my bowel movements (constipation, hemorrhoids)	3.0

*Wtd* weighted, *FIT* fecal immuochemical test, *CRC* colorectal cancer

Other reasons for not doing the FIT that were not in the original list included procrastination (7 %); a physical disability, e.g., limited use of an arm, tremor, arthritis, or poor eyesight that made it difficult to get the stool sample into the tube (1 %); hemorrhoids or severe constipation (3 %); fear of needing to have a colonoscopy if the FIT result was positive (3 %); not wanting to pay for the test, which was free (8 %); and having been screened outside of the health plan (1 %). The most frequent of these “other” responses are shown in Table 5. Blacks and Latinos and people ages 65–76 were significantly more likely than WhiteNHs and people ages 52–64, respectively, to indicate procrastination. Latinos were significantly more likely than WhiteNHs and Blacks (23.4 % vs. 9.1 %, *χ*^2^ = 3.9, *P* < .05) to indicate that they did not need to be screened because they felt fine.

Approximately 30 % of Nonusers had no interest in using the FIT kit. However, less than 10 % said they had absolutely no interest in being screened for CRC, and 41 % indicated that they had been screened for CRC in the past (21 % with a FOBT). Approximately 15 % said that they would consider doing the FIT or other type of CRC screening procedure if their doctor told them why it is important for *them* to get screened.

### Characteristics of the FIT kit that may be affecting FIT uptake and possible changes to the kit to boost uptake and continued use

**Fig. 1:**
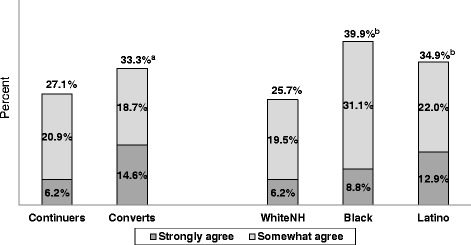
Percentages who agreed with statement: I am concerned about coming into contact with germs or bacteria in the toilet water or bowel movement”. ^a^Significantly greater than Continuers (*P* < .05, Wald chi-square test); ^b^Significantly greater than WhiteNH (*P* < .05, Wald chi-square test) after adjusting for age, sex, and study group or race/ethnicity

Nonusers were not the only people who felt uncomfortable about the procedures involved with using the FIT kit. Overall, 28 % of Continuers and Converts who did the FIT in 2012 strongly (7.0 %) or somewhat (20.7 %) agreed that they were concerned about coming into contact with germs/bacteria in the toilet water or bowel movement. As shown in Fig. 1, Converts were significantly more likely than Continuers, and Blacks and Latinos significantly more likely than WhiteNHs, to be concerned about coming into contact with germs or bacteria in the toilet water while getting their stool sample. Paralleling this concern, Converts were significantly more likely than Continuers (47.2 % vs. 26.5 %, *χ*^2^ = 13.6, *P* < .001, after adjusting for age, race/ethnicity, and sex), and Blacks were significantly more likely than WhiteNHs (40.5 % vs. 26.5 %, *χ*^2^ = 7.6, *P* < .01) to use their own disposable glove when doing the FIT. Further, when asked about changes they would recommend to the FIT kit to make it easier to use (Table 6), about 55 % of Continuers and Converts recommended including a disposable glove, with 27 % indicating that as their highest priority change. One-third of Continuers and Converts also recommended including an antibacterial wipe (highest priority change for 7 %).

**Table 6:** Changes to FIT kit recommended by people who used the FIT kit in 2012

Recommended Changes to FIT Kit	All	WhiteNH	Black	Latino
	(*N* = 799)	(*N* = 272)	(*N* = 252)	(*N* = 275)
	Wtd. %	Wtd. %	Wtd. %	Wtd. %
Include an extra sheet of paper	56.7	56.3	61.3	55.8
Include a disposable glove	54.7	51.8	68.7^*^	63.9^*^
Make tube mouth wider	46.9	45.2	58.9^*^	49.2
Include an antibacterial wipe	33.9	30.5	52.2 ^*^	44.0^*^
Make poke stick longer	19.7	17.6	29.3^*^	27.2^*^
Simplify the instructions	6.8^**^	6.3	9.1	8.8
Post a video demonstration of how to use the FIT kit	6.8	5.9	10.2	10.7

*Wtd* weighted, *WhiteNH* nonHispanic White, *FIT* fecal immunochemical test

^*^Significant (*P* < .05) difference compared to WhiteNH after adjusting for age group, sex, and study group

^**^Significant (*P* < .05) difference between Convert and Continuer group after adjusting for age group, sex, and race/ethnicity

Approximately 30 % of respondents who did the FIT in 2012 indicated that they sometimes had trouble catching their bowel movement sample on the paper provided, and 25 % said they sometimes had trouble getting the sample into the tube: 8.4 % disagreed and 21.9 % somewhat agreed with the statement “I have no trouble catching my bowel movement sample on the paper laid in the toilet” (the other category, “strongly agreed”, was interpreted as having no problem), and 5.6 % disagreed and 19.1 % somewhat agreed with the statement “I have no trouble getting the sample into the tube.” Paralleling these problems, over half of Continuers and Converts recommended including an extra sheet of paper (highest priority change for 32 %), 46 % requested a tube with a wider opening (highest priority change for 28 %), and 20 % wanted a longer poke stick (highest priority change for 5 %). From free-text comments, we surmised that the request for the extra sheet of paper is likely associated with misconception that the bowel movement sample becomes contaminated if it comes into contact with urine in the toilet bowl.

Blacks and Latinos were significantly more likely than WhiteNHs to want the disposable glove, an antibacterial wipe, and a longer poke stick, and Blacks were also more likely to request a wider-mouth tube. Women were significantly more likely than men (60.1 % vs. 47.4 %, *χ*^2^ = 4.4, *P* < .05) to request the inclusion of a glove. Continuers and Converts only differed significantly with regard to a request for simpler instructions (5.9 % vs. 14.7 %, respectively, *χ*^2^ = 10.2, *P* < .001).

Nonusers were also asked whether any of these changes would make them more likely to use the FIT kit in the future. Of the 117 who answered, the glove was indicated by 67 %, the extra sheet of paper and antibacterial wipe by 45 %, the longer poke stick and tube with wider mouth by 29 %, and simpler instructions by 10 %. There were no significant racial/ethnic or sex differences.

## Discussion

Our cross-sectional survey of three types of responders to a centralized FIT kit outreach program within an integrated health care program identified several differences in beliefs and concerns associated with FIT kit use between continuous users, people who had avoided doing the FIT for at least two years but did it in the outreach year studied, and people who continued to avoid doing it.

Among people who had been completing the FIT consistently for several years, the most frequently cited main motivations were intrinsic, i.e., “checking to make sure I’m OK” or “to protect my health.” As a group, they were more likely than non-routine FIT users to believe that it is very important for them to get screened for CRC. Among men, the doctor and spouse/partner (if in a relationship) was a major motivator for doing the FIT, but this was less true for women. Convenience of the FIT for CRC screening was a more important feature for WhiteNHs than for Blacks and Latinos.

Among people who did the FIT in 2012 after ignoring outreach efforts for at least two years, social influence (from their doctor or partner) and guilt were most frequently cited as reasons for doing it this time, with significantly lower percentages citing intrinsic motivations. However, Converts were less likely than Continuers to believe that it was very important to their medical care team and to themselves personally that they be screened. They were also more concerned than Continuers about handling their stool sample (e.g., they used a glove), less likely to indicate that the FIT was a convenient way to get CRC screening, and less likely to know that the FIT needs to be done annually to be effective for CRC screening. Those Converts who do not internalize the benefits of using the FIT for CRC screening or do not accept that it needs to be done annually are likely at elevated risk for not continuing to do the FIT annually.

Among people who have been avoiding doing the FIT, feeling uncomfortable or visceral disgust with the process of getting their stool sample out of the toilet and getting it into the tube is a major barrier to uptake. We suspect that many of those who only indicated procrastination as their reason for not doing the FIT were also avoiding it due to discomfort or embarrassment. For some, these feelings are based on past experience doing FOBTs, while for others, just the idea of doing the FIT is upsetting (e.g., one woman interviewed said she was so disgusted by the thought of doing the test that she got nauseous just talking about it). This disgust factor has also been found in previous studies [5, 8, 28, 30]. Based on what Nonusers told us, we think that many FIT avoiders are not convinced that the personal benefits of doing the FIT outweigh the costs (feelings of discomfort, disgust, or embarrassment related to preparing and sending their stool sample), especially if they think they are at low risk for CRC. Most people who have been avoiding doing the FIT did not say they are not interested in being screened for CRC, just that they did not like using a stool test.

We believe that the majority of people who have avoided doing the FIT for several years, despite outreach efforts, have developed resistance to the idea of doing the FIT that is not going to be overcome by different outreach messaging—they do not even want to think about or talk about doing a stool test. This is in keeping with Duncan *et al.* that the behavior of refusing multiple CRC screening offers is different from the behavior of refusing a single screening offer [33]. Evidence to support this comes from the fact that nearly 90 % of those in our Nonuser sample chose not to respond to two mailings of a short survey even when offered a $10 gift card to complete it, as compared with a 67 % response rate for people who had done the test every year for the past 3 years and a 36 % response rate for people who did the test in 2012 after 2 years of not doing it. Many of the 96 Nonusers who agreed to be interviewed expressed embarrassment about not using the FIT kit, but few indicated willingness to do it, and several expressed anger at feeling they were being pressured to use it.

Duncan *et al.* [33] found that level of satisfaction with past FOBT screening was a strong behavioral predictor of adherence, irregular use, or consistent refusal. Our study results suggest that rather than trying to increase uptake and annual use of the FIT by changing messaging or outreach strategies, it might be more productive to make relatively simple changes to the FIT kit and kit instructions that would make it easier and less unpleasant to use.

Physical changes to the kit might involve using a tube with a wider mouth and including a disposable glove and extra sheet of paper. Changes to the instructions might include a recommendation to increase water and fiber intake for a few days to make the bowel movement sample easier to get into the tube, and reassurance that the stool sample will not become contaminated if it comes into contact with toilet water or urine. To address confusion about when the FIT kit must be used, the instructions could mention the shelf life or provide a way for people to check to see if their kit is no longer usable. Labeling the return envelope “mail or drop off sample within X days” could decrease the number of people who prepare the sample but do not get it processed. Finally, given that over one-fourth of Converts and nearly 60 % of Nonusers did not realize that the FIT needs to be done every year, the reason why the FIT needs to be done annually may need to be communicated more effectively to those who have not established themselves as annual users.

Only about one-third of Nonusers who received FIT kits said that they opened them up, suggesting that changes made to the mailed kit may not result in greater uptake among this group unless they are alerted to the changes and given motivation to open it. Announcing improvements to the FIT kit on the outer envelope may be one way to do this.

It is likely that many Nonusers will need a more personal discussion with a health care provider about why it is important for them personally to get screened and, if they do not want to use the FIT, whether they would consider alternative methods, such as sigmoidoscopy or colonoscopy. A computer-generated letter purportedly coming from their primary care doctor and automated calls are obviously not motivating this group. A study by Wong *et al.* provides some evidence that allowing people to choose between the annual FIT and decennial colonoscopy after receiving a detailed description of what the latter entails may result in greater willingness to do the FIT annually than just being asked to do the FIT, even if it is an unpleasant experience [34]. Offering patients the opportunity to make an informed choice about CRC screening procedure may also address differences in race-ethnic preferences. Inadomi *et al.* found that offered the choice, Whites were more likely to prefer colonoscopy, while nonwhites were more likely to prefer FOBT [11]. In an accompanying editorial to the Inadomi *et al.* study, Levin discusses the importance of physician-patient communication and taking a more patient-center approach for increasing adherence to CRC screening recommendations [35].

Although we frequently saw sex differences in sources of influence and motivations for getting CRC screening, we did not see many racial/ethnic differences, suggesting that targeted messaging may not be needed for boosting FIT uptake in different segments of the population. Because Blacks and Latinos were more likely than WhiteNHs to express concern about exposure to germs/bacteria in the process of preparing their stool sample, inclusion of a glove and easier-to-use materials (extra paper if needed, tube with a wider opening) may improve the acceptability of the FIT kit to them.

Our study is limited by the low survey response rates for our Convert and Nonuser groups, despite the $10 incentive we offered. In addition to potential nonresponse bias, the low response rates also reduced our statistical power to assess racial/ethnic differences in factors that motivated or deterred use of the FIT kit within and across these study groups. A second limitation is that we have no information about nature of the relationships respondents had with their primary care physicians, nor the scope of the physician–patient interactions about the FIT and CRC screening among those who reported some type of discussion or recommendation. Another limitation, and perhaps most important in terms of interpreting the survey results, is that we have no information about our study sample’s CRC screening history outside of what was documented in their health plan records for calendar years 2010–2012. For example, we do not know what percentages of Converts and Nonusers in the original and respondent samples had never been screened for CRC using a blood stool test or other procedure. This means that both of these groups may be a mix of people who had never been screened for CRC, people who had been screened for CRC in the past but had not realized they needed to be screened again, and people who had a negative experience doing a blood stool test and decided to avoid doing the FIT. Also, some respondents in the Nonuser group told us that they were up to date with CRC screening, having had a colonoscopy done outside of the health plan.

Our study, however, also has several strengths. It focused on factors influencing uptake of one of the newer, less costly, and less invasive CRC screening tests. Most of the previous studies of barriers to CRC screening have focused on barriers to more invasive procedures (colonoscopy and sigmoidoscopy), CRC screening by any method, or multiple-sample FOBT that often involves dietary restrictions [4–9, 21–24] rather than on the easier 1-sample FIT with no dietary restrictions. Another strength is that we examined differences in social influences, beliefs, and characteristics of the FIT kit that motivate use and non-use of the one-sample FIT kit. This was done in the context of a centralized CRC screening outreach program within a large health care delivery system that included offering the FIT for free, mailing the kits to members’ homes, and formal encouragement to complete the FIT from the member’s primary care physician via a separately mailed letter. Most of these prior studies also drew from populations with different levels of financial coverage for CRC screening and differential outreach strategies for CRC screening. Finally, our data on patient-recommended changes to the standard FIT kit and to identify gaps in knowledge about how to use the FIT kit that might lead to making doing the test an easier and a less unpleasant experience.

## Conclusions

This study found that, compared with continuous FIT users, nonusers and irregular users of the FIT are less intrinsically motivated to get CRC screening and more averse to preparing their stool sample. Feelings of discomfort, disgust, and embarrassment with collecting the stool are the major reasons for lack of uptake indicated by nonusers. Future controlled trials could show whether changes to the FIT kit to address these factors might improve uptake and continuous use.

## Additional files

Additional file 1:
**FIT Kit Survey Questionnaires.** This is a pdf file containing both the Continuer/Convert questionnaire and Nonuser questionnaire. Additional file 2: Table S1. Response rates for each Study Group by race-ethnicity and age. this is a pdf file. Additional file 3: Figure S1. Perceived risk of developing colorectal cancer. CRC, colorectal cancer. This is a pdf file. Additional file 4: Figure S2. Responses to the question “How important do you think it is to your medical care team that you get screened for colorectal cancer?” This is a pdf file. Additional file 5: Figure S3. Responses to the question “How important do you think it is for you to get screened for colorectal cancer?” This is a pdf file.
