# Supplementary material for: Factors associated with use and non-use of the Fecal Immunochemical Test (FIT) kit for Colorectal Cancer Screening in Response to a 2012 outreach screening program: a survey study
Source: BMC Public Health. 2015 Jun 11;15:546. doi: 10.1186/s12889-015-1908-x (PMC4462185; doi:10.1186/s12889-015-1908-x)
Supplement: Additional file 1: — FIT Kit Survey Questionnaires. This is a pdf file containing both the Continuer/Convert questionnaire and Nonuser questionnaire. [file 12889_2015_1908_MOESM1_ESM.pdf]

Survey questionnaire used for Continuers and Converts

KAISER PERMANENTE®

DIVISION OF RESEARCH
Northern California

PLEASE ANSWER THESE QUESTIONS TO HELP KAISER PERMANENTE IMPROVE OUR COLORECTAL CANCER (CRC) SCREENING SERVICES

(AND WE'LL SEND YOU A $10 GIFT CARD)

The FIT (Fecal Immunochemical Test) is a test used to screen for early signs of colorectal cancer (CRC), also known as bowel, colon, or rectal cancer The FIT checks for blood in your bowel movement ("poop") from bleeding polyps (small growths) in the colon. These colon polyps can then be removed, usually before they become cancerous. Kaiser Permanente sends members FIT kits so that they can get the bowel movement sample at home and mail it into the lab for testing.

Our records show that in 2012, you completed a FIT colon cancer screening test. Congratulations on taking that step to try to maintain your health!

We want to learn about your situation and things that may have influenced you to use this CRC screening procedure. We also want to hear your ideas for changes that would make the FIT kit easier to use.

After you fill out and return this short questionnaire or complete it online, we will send you a $10 gift card for Target, Safeway/Pak 'n Save, Lucky/FoodMaxx, or Starbucks. Your answers will be kept confidential. They will not go into your medical record or be shared with anyone outside the Division of Research in a way that could identify you.

Please send your completed questionnaire to
Kaiser Permanente Division of Research
2000 Broadway
Oakland, CA 94612
ATTN: NPG

No stamp is necessary if you use the pre-addressed postage-paid envelope enclosed.

If you need assistance, please contact [PI email address] or call our toll-free line (800) XXX-XXXX.

Thank you.

Nancy P. Gordon

Nancy P. Gordon, ScD
Survey Director

1. Did you receive more than one FIT kit in 2012? *(Mark **ALL** that apply)*

☐ I got one FIT kit in the mail
☐ I got more than one FIT kit in the mail
☐ I got a FIT kit from my doctor or doctor's staff (at an office visit or sent with a personal note)
☐ I got a FIT kit when I came in for a flu shot

2. Did your doctor recommend that you do the FIT colorectal cancer screening test? If so, how? *(Mark **ALL** that apply)*

☐ My doctor told me (in person, by phone, by email, or in a handwritten note) to do this test
☐ I was sent a letter from my doctor recommending that I do the test
☐ My doctor had his medical office staff give me/send me a FIT kit
☐ My doctor talked with me about using the FIT test during an office visit or phone visit
☐ I have not talked with my doctor about colorectal cancer screening
☐ Other:____

3. Have any of the following people encouraged you to get screened for colorectal cancer? *(Mark **ALL** that apply)*

☐ Your spouse/partner
☐ Your children or grandchildren
☐ Other relatives
☐ Friends or co-workers
☐ Your employer
☐ Your minister, priest or rabbi

4. How important do you think it is to your Kaiser Permanente medical care team that you get screened for colorectal cancer?

☐ Not important ☐ Somewhat important ☐ Moderately important ☐ Very important

5. How important do <u>you</u> think it is for you to get screened for colorectal cancer?

☐ Not important ☐ Somewhat important ☐ Moderately important ☐ Very important

6. Which of these reasons influenced you to use the FIT kit in 2012? *(Mark **ALL** that apply)*

☐ My doctor really wanted me to do the FIT test
☐ My spouse/partner really wanted me to do it
☐ My children or grandchildren really wanted me to do it
☐ My employer really wanted me to do it

☐ I received several FIT kits and began to feel guilty about not doing the test
☐ I have a family history of colon polyps or colorectal cancer
☐ I personally know someone who had pre-cancerous colon polyps or colon (bowel) cancer
☐ I have been diagnosed with another type of cancer
☐ I wanted to make sure that I was OK

☐ The FIT test is free
☐ The FIT test is more convenient than other colorectal cancer screening methods
☐ The FIT test needs to be done every year

☐ Having the FIT test can help me protect my health
☐ When colorectal polyps are found and removed, colorectal cancer can be prevented
☐ Other:____

If you marked <u>more than 2</u> reasons, please put stars (★) next to your <u>top 2 reasons</u>.

7. What do you think are your chances of getting:

	Very low	Low	Medium	High
**a. Colon polyps (small growths that can become cancerous)?**	☐	☐	☐	☐
**b. Cancer of the colon or rectum?**	☐	☐	☐	☐

8. Please tell us whether you agree with each of the following statements.

	Strongly Agree	Somewhat Agree	Don't Agree
**a. I think the FIT kit instructions are easy to follow.**	☐	☐	☐
**b. I have no trouble catching the bowel movement sample on the paper.**	☐	☐	☐
**c. I have no trouble getting the sample into the tube.**	☐	☐	☐
**d. I am concerned about coming into contact with germs and bacteria in the toilet water or my bowel movement.**	☐	☐	☐
**e. The FIT kit is a convenient way to be screened for colon polyps and colorectal cancer.**	☐	☐	☐

9. To get your bowel movement sample, do you use the method described in the FIT kit instructions? ☐ Yes ☐ No

10. When you use the FIT kit, do you use a disposable glove? ☐ Yes ☐ No

11. Did you know that Kaiser Permanente Northern California members can get the FIT done for free? ☐ Yes ☐ No

12. Did you know that the FIT needs to be done every year to be effective? ☐ Yes ☐ No

13. While it is best to use the FIT kit within a day or two of receiving it, how long do you think you have to use it before it goes bad?

☐ 2 weeks ☐ 4 weeks ☐ 6 weeks ☐ 8 weeks ☐ 3 months

14. While it is best to mail your FIT sample back to the lab within a day of putting it in the test tube, when is the <u>latest</u> you think it can be mailed and still get valid results?

☐ 3 days after ☐ 5 days after ☐ 7 days after ☐ 10 days after

15. Which of the following changes would you like us to consider for the FIT kit?
*(Mark **ALL** that apply and write in other suggestions)*

☐ Include a disposable glove in the FIT kit
☐ Include an antibacterial wipe in the FIT kit
☐ Include an extra piece of paper to lay in the toilet in case I have problems the first time
☐ Use a longer stick in the FIT kit to scoop up the bowel movement sample
☐ Use a tube with a wider opening in the FIT kit so it is easier to get the sample inside
☐ Make the FIT kit instructions easier to follow (for example, use more pictures)
☐ Post a video on YouTube or the kp.org website so people can watch how to use the FIT kit
☐ Other: ____

If you marked more than 1, please put a star (★) next to the 1 change you would most like to see.

16. How would you prefer to get an alert that you are due for your annual FIT test?
*(Mark **ALL** that apply)*

☐ A mailed letter from my doctor (like you get now)
☐ A secure message (email) from my doctor (requires logging onto kp.org)
☐ A secure message (email) from someone in my doctor's office (requires logging onto kp.org)
☐ A text message
☐ An automated phone message

About you

17. Your sex: ☐ Male ☐ Female

18. Your age: _______

19. What is the <u>highest</u> level of education you have completed?

☐ 8th grade or less
☐ Some high school
☐ High school graduate or GED
☐ Some college or Associates (AA) degree
☐ College graduate (*B.A., B.S., etc*.)
☐ Graduate or professional degree

20. Are you currently married or in a committed relationship? ☐ Yes ☐ No

21. Which of the following is true of you? *(Mark **ALL** that apply)*

☐ I have a family history of pre-cancerous colon polyps or colon (bowel) cancer
☐ I have been diagnosed with another type of cancer
☐ I personally know someone who had pre-cancerous colon polyps or colon (bowel) cancer
☐ I have had irritable bowel syndrome (IBS), ulcerative colitis, or Crohn's disease
☐ I eat a lot of red meat (steak, beef, hamburger)
☐ I eat a lot of foods high in fat ("fast foods", fried foods, foods with a lot of butter or margarine, cookies, cake, pie, or chocolate candy)
☐ I eat a lot of high-fiber foods (whole grains, fresh fruits/vegetables) or take a fiber supplement
☐ I take a low dose aspirin daily

Which $10 gift card would you prefer? *(Mark **ALL** that would be acceptable)*

☐ Target ☐ Safeway/Pak 'n Save ☐ Lucky/FoodMaxx ☐ Starbucks

Thank you for completing this questionnaire.
Please mail it to the address on the front cover.

Survey questionnaire used for Nonusers

KAISER PERMANENTE®

DIVISION OF RESEARCH
Northern California

PLEASE ANSWER THESE QUESTIONS TO HELP KAISER PERMANENTE IMPROVE OUR COLORECTAL CANCER (CRC) SCREENING SERVICES

(AND WE'LL SEND YOU A $10 GIFT CARD)

The FIT (Fecal Immunochemical Test) is used to screen for early signs of colorectal cancer (CRC), also known as bowel, colon, or rectal cancer. The FIT checks for blood in your bowel movement ("poop") from bleeding polyps (small growths) in the colon. These colon polyps can then be removed, usually before they become cancerous. Kaiser Permanente sends members FIT kits so that they can get the bowel movement sample at home and mail it into the lab for testing.

Our records show that in 2012, you were sent a FIT kit but did not send your sample to the lab. We want to learn about your situation and things that may have influenced your decision about using the FIT kit. We also want to hear your ideas for changes that could make the FIT kit easier to use.

After you fill out and return this short questionnaire or complete it online, we will send you a $10 gift card for Target, Safeway/Pak 'n Save, Lucky/FoodMaxx, or Starbucks. Your answers will be kept confidential. They will not go into your medical record or be shared with anyone outside the Division of Research in a way that could identify you.

Please send your completed questionnaire to:
Kaiser Permanente Division of Research
2000 Broadway
Oakland, CA 94612
ATTN: NPG

No stamp is necessary if you use the pre-addressed, postage-paid envelope enclosed.

If you need assistance, please contact [PI email address] or call our toll-free line 800-XXX-XXXX.

Thank you.

Nancy P. Gordon

Nancy P. Gordon, ScD
Survey Director

1. **Do you remember receiving one or more FIT kits in 2012?** *(Mark **ALL** that apply)*
   ☐ I don't remember getting a FIT kit
   ☐ I got one FIT kit in the mail
   ☐ I got more than one FIT kit in the mail
   ☐ I got a FIT kit from my doctor or doctor's staff (at an office visit or sent with a personal note)
   ☐ I got a FIT kit when I came in for a flu shot

2. **Did you read the letter from your doctor telling you the FIT kit was coming?** ☐ Yes ☐ No

3. **When you received your FIT kit, did you open it and read the instructions?** ☐ Yes ☐ No

4. **Did your doctor recommend that you do the FIT colorectal cancer screening test? If so, how?** *(Mark **ALL** that apply)*
   ☐ My doctor told me (in person, by phone, by email, or in a handwritten note) to do the test
   ☐ I was sent a letter from my doctor recommending that I do the test
   ☐ My doctor had his medical office staff give me/send me a FIT kit
   ☐ My doctor talked with me about the FIT test during an office visit or phone visit
   ☐ My doctor has told me that I should get screened for colorectal cancer, but we haven't discussed which method to use
   ☐ I have not talked with my doctor about colorectal cancer screening
   ☐ Other:____

5. **Have any of the following people encouraged you to get screened for colorectal cancer?** *(Mark **ALL** that apply)*
   ☐ Your spouse/partner
   ☐ Your children or grandchildren
   ☐ Other relatives
   ☐ Friends or co-workers
   ☐ Your employer
   ☐ Your minister, priest or rabbi

6. **How important do you think it is to your Kaiser Permanente medical care team that you get screened for colorectal cancer?**
   ☐ Not important ☐ Somewhat important ☐ Moderately important ☐ Very important

7. **How important do <u>you</u> think it is for you to get screened for colorectal cancer?**
   ☐ Not important ☐ Somewhat important ☐ Moderately important ☐ Very important

8. **What do you think are your chances of getting:**

	Very low	Low	Medium	High
**a. Colon polyps (small growths that can become cancerous)?**	☐	☐	☐	☐
**b. Cancer of the colon or rectum?**	☐	☐	☐	☐

9. People have different reasons for not using the FIT kit. <u>Please mark the box next to each reason that applies to you</u>. You can also write in other reasons.

I have problems with using the FIT kit:

☐ The idea of doing this test involving my bowel movement (“poop”) makes me uncomfortable
☐ I feel disgusted by the idea of reaching into the toilet to get a sample of my bowel movement
☐ I am concerned about coming into contact with germs and bacteria
☐ It’s too messy to do this test
☐ I think it will be too hard for me to get the bowel movement sample and put it in the tube
☐ I am embarrassed to put the FIT kit by the toilet, but then I keep forgetting to use it
☐ I tried to scoop up a bit of the bowel movement and put it into the tube, but it was too hard to do
☐ I used the FIT kit to get the sample, but then forgot to put the envelope in the mail right away
☐ I don’t like the idea of sending my bowel movement sample through the mail
☐ I don’t think that the FIT test is effective for finding cancer early
☐ Other:____

I don’t want to do the FIT test because:

☐ I feel fine, so why look for trouble
☐ I think my chances of developing colorectal cancer are very low
☐ I think if I am meant to get colon cancer, I will get it no matter what I do
☐ I think that even if colon cancer is detected early, nothing can be done about it
☐ I don’t want to have to worry until I get the results of the test
☐ If I have colon cancer, I don’t want to know
☐ I don’t want to have to pay for this test
☐ Other: ____

If you marked <u>more than 2</u> reasons, please put a star (*) next to your <u>top 2 reasons</u>.

10. Have you <u>ever</u> had any of these colorectal cancer screening procedures in the past?
*(Mark **ALL** tests you’ve had)*

☐ FIT (Fecal Immunochemical Test)
☐ FOBT (test used before the FIT to check for blood in the bowel movement)
☐ Sigmoidoscopy (flexible tube inserted through the rectum or “butt hole” to look for colon polyps)
☐ Colonoscopy (procedure to search the whole colon to look for polyps; requires anesthesia)

11. Did you know that Kaiser Permanente Northern California members can get the FIT done for free? ☐ Yes ☐ No

12. Did you know that the FIT needs to be done every year to be effective? ☐ Yes ☐ No

13. While it is best to use the FIT kit within a day or two of receiving it, how long do you think you have to use it before it goes bad?

☐ 2 weeks ☐ 4 weeks ☐ 6 weeks ☐ 8 weeks ☐ 3 months

14. While it is best to mail your FIT sample back to the lab within a day of putting it in the test tube, when is the <u>latest</u> you think it can be mailed and still get valid results?

☐ 3 days after ☐ 5 days after ☐ 7 days after ☐ 10 days after

15. Which of the following would make you more likely to use the FIT kit in the future? (*Mark **ALL** that apply and write in other suggestions)*

☐ My doctor needs to tell me why it is important <u>for me</u> to have this screening test
☐ Include a disposable glove in the FIT kit
☐ Include an antibacterial wipe in the FIT kit
☐ Include an extra piece of paper to lay in the toilet in case I have problems the first time
☐ Use a longer stick in the FIT kit to scoop up the bowel movement sample
☐ Use a tube with a wider opening in the FIT kit so it is easier to get the sample inside
☐ Make the FIT kit instructions easier to follow (for example, use more pictures)
☐ Post a video on YouTube or the kp.org website so I can watch how to use the FIT kit
☐ Other: ____
☐ Nothing would make me more likely to use the FIT kit

If you marked <u>more than 1</u> suggestion, please put a star (*) next to the <u>1 change that would influence you the most</u> to use the FIT kit in the future.

16. If you definitely <u>do not</u> want to use the FIT kit but would be open to other methods for colon cancer screening, how would you prefer to learn about them? *(Mark **ALL** that apply)*

☐ Have my doctor talk to me about the other methods in person or by phone
☐ Have my doctor send me an email recommending what other methods I should consider
☐ Send me an email link to information about alternatives on the kp.org website
☐ Send me print materials in the mail about these alternatives
☐ I am not interested in getting screened for colorectal cancer at this time

About you

17. Your sex: ☐ Male ☐ Female

18. Your age: _______

19. What is the <u>highest</u> level of education you have completed?

☐ 8th grade or less
☐ Some high school
☐ High school graduate or GED
☐ Some college or Associates (AA) degree
☐ College graduate (*B.A., B.S., etc*.)
☐ Graduate or professional degree

20. Are you currently married or in a committed relationship? ☐ Yes ☐ No

21. Which of the following is true of you? *(Mark **ALL** that apply)*

☐ I have a family history of pre-cancerous colon polyps or colon (bowel) cancer
☐ I have been diagnosed with another type of cancer
☐ I personally know someone who had pre-cancerous colon polyps or colon (bowel) cancer
☐ I have had irritable bowel syndrome (IBS), ulcerative colitis, or Crohn's disease
☐ I eat a lot of red meat (steak, beef, hamburger)
☐ I eat a lot of foods high in fat ("fast foods," fried foods, foods with a lot of butter or margarine, cookies, cake, pie, or chocolate candy)
☐ I eat a lot of high-fiber foods (whole grains, fresh fruits/vegetables) or take a fiber supplement
☐ I take a low dose aspirin daily

Which $10 gift card would you prefer? *(Mark **ALL** that would be acceptable)*

☐ Target ☐ Safeway/Pak 'n Save ☐ Lucky/FoodMaxx ☐ Starbucks

Thank you for completing this questionnaire. Please mail it to the address on the front cover.
