# Supplementary material for: Factors associated with use and non-use of the Fecal Immunochemical Test (FIT) kit for Colorectal Cancer Screening in Response to a 2012 outreach screening program: a survey study
Source: BMC Public Health. 2015 Jun 11;15:546. doi: 10.1186/s12889-015-1908-x (PMC4462185; doi:10.1186/s12889-015-1908-x)
Supplement: Additional file 2: Table S1. — Response rates for each Study Group by race-ethnicity and age. this is a pdf file. [file 12889_2015_1908_MOESM2_ESM.pdf]

Response to the FIT kit survey for study groups by race-ethnicity and age

	Continuers	Converts	Nonusers
White			
All	73.7% (221/300)	41.3% (124/300)	21.8% (74/339)
52-64	75.3% (113/150)	41.3% (62/150)	18.8% (32/170)
65-76	72.0% (108/150)	41.3% (62/150)	24.8% (42/169)
Black			
All	60.9% (182/299)	32.4% (94/290)	19.8% (67/338)
52-64	59.7% (89/149)	34.9% (52/149)	15.5% (26/168)
65-76	62.0% (93/150)	29.8% (42/141)	24.1% (41/170)
Latino			
All	68.0% (204/300)	33.0% (99/300)	21.8% (74/340)
52-64	66.0% (99/150)	34.0% (51/150)	24.1% (41/170)
65-76	70.0% (105/150)	32.0% (48/150)	19.4% (33/170)
