# Supplementary material for: Factors associated with use and non-use of the Fecal Immunochemical Test (FIT) kit for Colorectal Cancer Screening in Response to a 2012 outreach screening program: a survey study
Source: BMC Public Health. 2015 Jun 11;15:546. doi: 10.1186/s12889-015-1908-x (PMC4462185; doi:10.1186/s12889-015-1908-x)
Supplement: Additional file 3: Figure S1. — Perceived risk of developing colorectal cancer. CRC, colorectal cancer. This is a pdf file. [file 12889_2015_1908_MOESM3_ESM.pptx]

## Slide 1
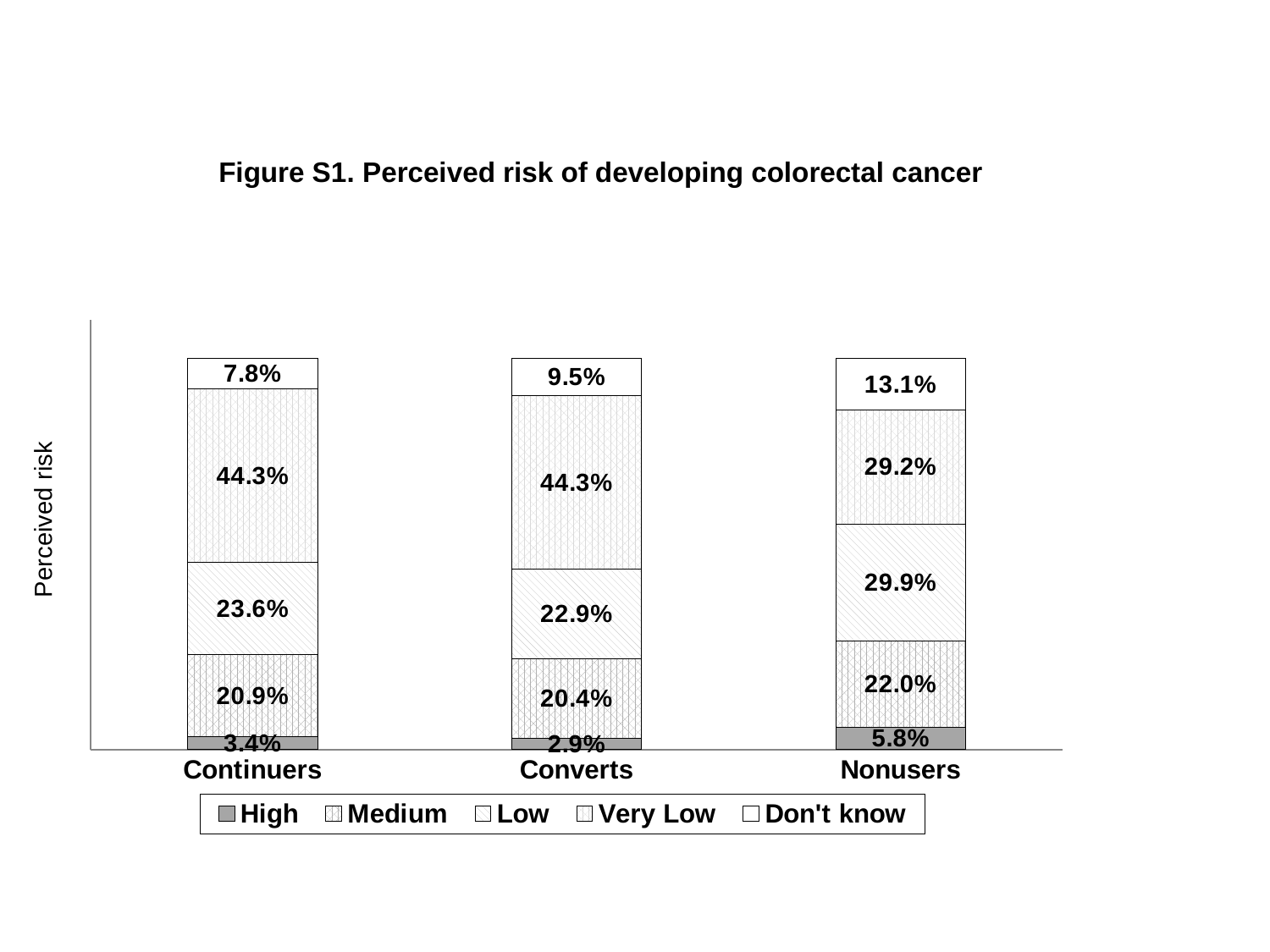

#
Figure S1. Perceived risk of developing colorectal cancer
### Chart
| Category | High | Medium | Low | Very Low | Don't know |
|---|---|---|---|---|---|
| Continuers | 0.034 | 0.209 | 0.236 | 0.443 | 0.078 |
| Converts | 0.029 | 0.204 | 0.229 | 0.443 | 0.095 |
| Nonusers | 0.058 | 0.22 | 0.299 | 0.292 | 0.131 |Perceived risk
