# Supplementary material for: Factors associated with use and non-use of the Fecal Immunochemical Test (FIT) kit for Colorectal Cancer Screening in Response to a 2012 outreach screening program: a survey study
Source: BMC Public Health. 2015 Jun 11;15:546. doi: 10.1186/s12889-015-1908-x (PMC4462185; doi:10.1186/s12889-015-1908-x)

Figure S3. Responses to the question "How important do you think it is for you to get screened for colorectal cancer?"

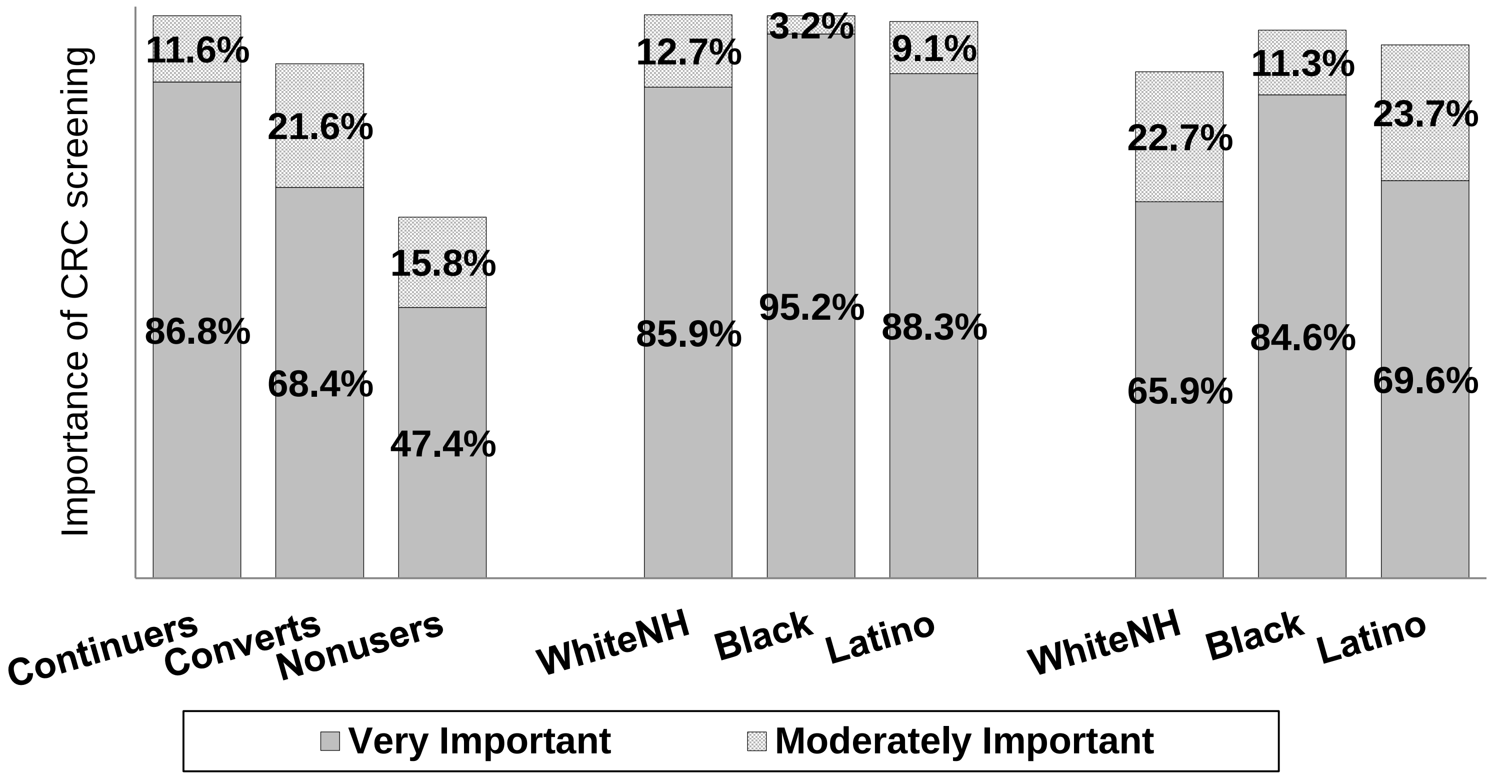

Supplement: Additional file 5: Figure S3. — Responses to the question “How important do you think it is for you to get screened for colorectal cancer?” This is a pdf file. [file 12889_2015_1908_MOESM5_ESM.pdf]
